# Prevalence and Incidence of Upper Respiratory Tract Infection Events Are Elevated Prior to the Development of Rheumatoid Arthritis in First-Degree Relatives

**DOI:** 10.3389/fimmu.2018.02771

**Published:** 2018-11-29

**Authors:** Marina I. Arleevskaya, Shafigullina Albina, Regina V. Larionova, Aida G. Gabdoulkhakova, Julie Lemerle, Yves Renaudineau

**Affiliations:** ^1^Central Research Laboratory, State Medical Academy, Kazan, Russia; ^2^Laboratory of Immunology and Immunotherapy, INSERM U1227, Hôpital Morvan, Centre Hospitalier Regional Universitaire de Brest, Brest, France

**Keywords:** rheumatoid arthritis, infections, first-degree relatives, upper respiratory tract infection symptoms, herpes virus

## Abstract

**Introduction:** The aim of this study was to characterize infection events in a longitudinal cohort of first-degree relatives (FDR) of probands with rheumatoid arthritis (RA) and explore their associations with RA development. To this end, newly diagnosed RA patients (*n* = 283), unaffected related FDR and age-matched healthy women were ascertained from the Caucasian triple women prospective Tatarstan cohort.

**Methods:** In this cohort initiated in 1997, 26/283 (9.2%) FDR developed RA (incidence: 9.1 cases/1,000/year). At baseline and during the follow-up, information regarding infectious events (prevalence) and their incidence and duration per year were collected from all individuals.

**Results:** Results reveal in the unaffected FDR developing RA subgroup: (i) a higher prevalence and/or incidence at baseline of upper respiratory infections (URI), otitis, tonsillitis, herpes reactivation, and skin infections; (ii) *Mycoplasma sp* detection was increased during pregnancy; (iii) a peak of infections started in the 3 years preceding RA onset, and thereafter decreased following RA diagnosis and treatment initiation with disease-modifying anti-rheumatic drugs (DMARDs) when considering URI, and acute tonsillitis; (iv) herpes virus reactivation, at baseline, was associated with a higher report of morning stiffness and arthralgia while independent from rheumatoid factors and anti-citrullinated peptide (CCP)2 Ab positivity; and (v) infection events represent an independent environmental factor associated with RA development.

**Conclusion:** In conclusion, an annual increase of respiratory tract infections was found at the pre-clinical stage of RA. This could be due to alterations in the immune system that result in susceptibility to infection, controlled by DMARDs, or that the infectious events predispose to RA.

## Introduction

Infections have been considered as important risk factors associated with the development of rheumatoid arthritis (RA) and mechanisms are related to molecular mimicry as reported for cytomegalovirus (CMV), Epstein-Barr virus (EBV) and parvovirus B19 (1); to citrullinated peptides derived from viruses and bacteria as the conversion of arginine to citrulline increases peptide binding affinity to the RA associated HLA-DRB1 shared epitope (2); and to latent viruses and intracellular bacteria present in fibroblast-like synoviocytes (FLS) and self-reactive memory B cells from RA patients (3). The infection hypothesis supporting infection as being a trigger for RA is further supported by the reports of a higher frequency of antibody (Ab) against the human herpes family (HSV1/2, EBV, and CMV), parvovirus B19, *Mycoplasma sp* as well as chikungunya virus (4–6). In addition, viral reactivation following biotherapies has been reported (7). Altogether, these arguments support a causative role for infections in RA development. However, these findings regarding the link between Ab against infections and RA risk have not been replicated in large-scale cohorts and meta-analysis (8, 9), leading instead to the proposition of an immunological hypothesis as one of the characteristics of RA patients in whom there is an inability of the immune system to properly control viral infections (10). This is in line with our recent findings, performed at the early stage of RA development in untreated subjects, showing both defects in the innate immune system and an elevated annual rate of infection events (11, 12).

In susceptible individuals, such as first-degree relatives (FDR) from probands with RA, interactions between environmental factors, including infections, and genetic risk factors are suspected to lead to preclinical symptoms and autoimmunity. This state is characterized by joint symptoms (13), a proinflammatory status (14), and early detection of RA-related autoantibodies (Ab) including rheumatoid factor (RF), and anti-cyclic citrullinated peptide (CCP) antibodies (Ab) that develop months to years prior to the onset of clinically evident synovitis (15). Epidemiologic studies have further established a long list of genetic and non-infectious environmental risk factors for RA, such as smoking, excess body mass index (BMI), and education level but also protective factors, such as low to moderate alcohol consumption, and fish consumption as a source of omega-3 (16–20). As such, studying the FDR population represents an ideal model to further elucidate mechanisms of RA pathogenesis that are not completely understood.

In order to characterize infections during RA development at pre-clinical stage, the 20-years longitudinal Tatarstan women cohort was selected. In this prospective cohort, three groups were invited to participate: (i) women with newly diagnosed RA (< 1 year after RA diagnosis); (ii) their unaffected FDR and, from these, those developing RA during the follow-up were analyzed; and (iii) FDR age-matched control women without a personal or family history of RA. At baseline and during the follow-up, total and individual infection characteristics regarding their annual prevalence, incidence and event duration were evaluated. An increase in the annual prevalence and/or incidence of infection events was found in the FDR subgroup developing RA and this then is an independent factor associated with RA development at the pre-clinical stage in FDR women from probands with RA.

## Materials and methods

### Subjects

In this prospective, matched, triple cohort conducted in Caucasian women between 1997 and 2017 at the Kazan State Medical Academy, the subjects were newly diagnosed (< 1 year) patients with RA (*n* = 283, median age 51 years [IQR: 41–60]) including 63/283 (22.3%) who have initiated treatment with disease-modifying anti-rheumatic drugs (DMARDs), FDR (*n* = 283), and 283 age-matched healthy controls. Healthy controls included subjects without any signs of chronic disease, and no RA among close relatives. Allergic disease with detection of allergen-specific IgE, and positivity for human immunodeficiency virus (HIV) in the history were considered as exclusionary criterion. RA diagnosis was established according to the 2010 ACR/EULAR classification criteria (21), and for patients diagnosed before 2010 by using a consensus of three experienced rheumatologists. In this study, the authors estimated a 60% capture rate of new RA patients in the cohort. The study was approved by the Ethical Committee of the Kazan State Medical Academy, Kazan, Russia (Permit nr 1/2002). Consent was received from all the individuals involved in the study, including consent to participate in the study and consent to allow publication of the results.

At baseline (i.e., at study entry), a questionnaire was filled out dealing with epidemiologic and environmental factors associated with RA: infections (see below), age, cigarette smoking, alcohol intake, BMI, education and fish consumption. For cigarette smoking and since the consumption was limited in the studied population, we categorized patients as non-smoker, passive smoker or active smoker. For the same reason, alcohol was categorized as no consumption, rare consumption or regularly consumes. Education was categorized as secondary graduate, high school graduate, or university graduate. In addition baseline characteristics of the study population included demographic data; joint symptoms evaluation by a rheumatologist (AMI): arthralgia in previous year, morning stiffness >30 min in the previous year, non-erosive arthritis previously in their life, and joint symptoms previously in their life. RF and anti-CCP2 autoantibodies were categorized into three groups based on the RF or anti-CCP2 Ab cut-off levels: negative, intermediate, and high (3 times the reference cut-off level) as defined in the 2010 RA classification criteria (21).

An annual and optional follow-up was proposed for both FDR subjects and healthy controls, performed by a rheumatologist (AMI) and the evaluation was based on answers to the infectious questionnaire and joint symptoms evaluation. In case of joint symptoms (pain and morning stiffness) in the small joints of the feet and hands, subjects underwent magnetic resonance imaging (MRI). The laboratory assays were repeated if necessary. During this 20-years follow-up, 26 FDR subjects have developed RA.

### Infectious history

At baseline and during the follow-up, information on the history of infections was obtained from individuals as they were asked about the number of total or individual episodes of infections and their overall duration per year. Next, a physician qualified in rheumatology (AMI) checked the questionnaire with the individuals to validate (or not) the criteria defined in Supplementary Table 1 and information were retrospectively completed by exploring the outpatient medical records.

The following parameters of the infectious syndrome were analyzed: no infection, viral-suspected upper respiratory tract infection symptoms (URI), bacterial-suspected URI requiring antibiotic therapy, HSV exacerbation/reactivation, herpes zoster, acute and chronic exacerbation of tonsillitis/sinusitis/bronchitis, otitis, pneumonia, upper and lower urinary tract infection, stomatitis, and skin infection.

In addition, information collected from the outpatient medical records at inclusion or during the follow-up from the medical registry included human viral hepatitis status (HAV, HBV, and HCV), and *Mycobacterium tuberculosis* infection history and when an infection was reported or suspected the gamma interferon release assay was performed (Quantiferon-TB, Qiagen). Information regarding genital *Mycoplasma species* (chlamydia, mycoplasma, and ureaplasma) infection was collected at pregnancy.

### Statistical analysis

The Fisher's exact test was used for categorical data, continuous data are described as median and interquartile (IQR) and analyzed by one-way non-parametric ANOVA (Kruskal-Wallis). The results were corrected for global infections using the Dunn's Test and regarding the multiple infections using the false discovery rate online calculator for multiple testing based on the Benjamini–Hochberg method and *p*-values under 0.05 were considered significant (https://www.sdmproject.com/utilities/?show=FDR). Statistical analyses and heat map were performed using GraphPad Prism 7.0a (La Jolla, CA).

## Results

### Cohort description

**Figure 1:**
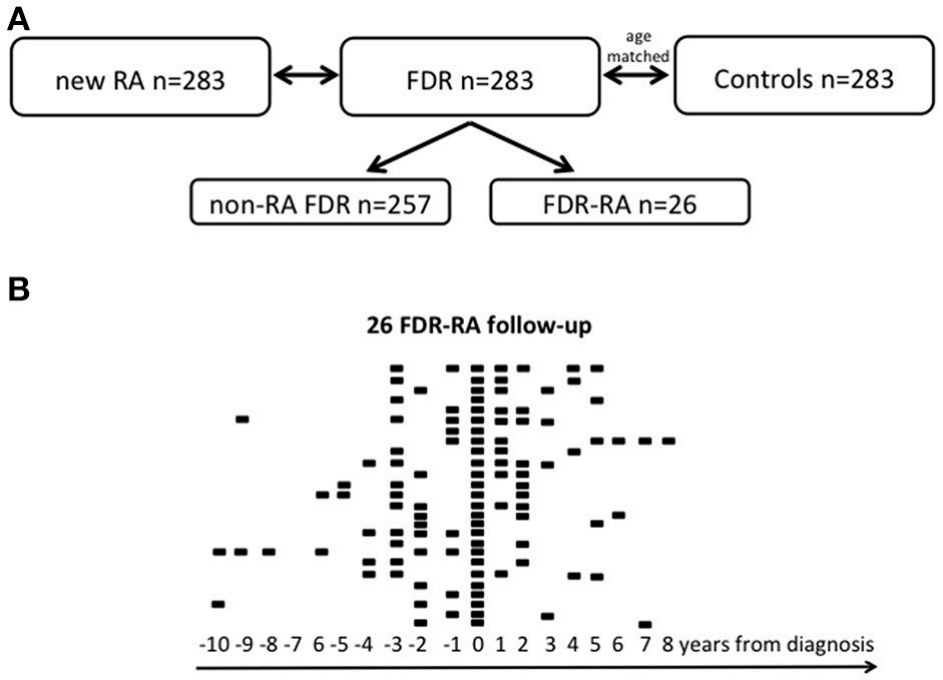
Study cohort. **(A)** The 20-year longitudinal Tatarstan women cohort is subdivided in three groups: women with newly diagnosed rheumatoid arthritis (< 1 year from diagnosis, new RA), unaffected first degree relatives (FDR), and healthy controls that are age matched with FDR. Among FDR, a subgroup has developed RA during its follow-up and was referred as FDR-RA in contrast to non-RA FDR who have not developed RA. **(B)** Follow-up of the 26 FDR having developed RA and for each individual available data are indicated according to the year of diagnosis (time = 0). *n*, number.

A total of 849 women were included in this prospective triple cohort study. Newly diagnosed RA patients (*n* = 283) were associated with their FDR, and with age-matched healthy controls (Figure 1A). During the 20 years cohort follow-up, the number of FDR who developed RA was 26 corresponding to 9.1 cases/1,000/year if considering a median duration follow-up of 9.0 years. For the FDR developing RA subgroup (FDR-RA), time from enrollment to RA diagnosis was 38.4 months (range: 12–123 months) and available data during their follow-up is depicted in Figure 1B. With the objective to study episodes of infection associated with RA development, a questionnaire was administered at baseline in order to evaluate episodes of infection in the previous year. The questionnaire evaluation was made with the subject by a rheumatologist, and if necessary information were retrospectively completed by exploring the outpatient medical records (see Supplementary Table 1).

Next, to delve into the episodes of infection within the previous year, the 26 FDR developing RA, the 257 non-RA FDR, the 283 controls, and the 283 newly diagnosed RA patients were compared. For each specific type of infection, collected data included (i) annual infection prevalence (%); (ii) the total number of infection events per year (incidence), and (iii) the episode duration per year for each specific type of infection in the subject's history (Table 1). In addition, the prevalence for viral hepatitis, tuberculosis, and *Mycoplasma* sp. contamination during pregnancy were also collected.

**Table 1:** Infection event characteristics at baseline in healthy controls, in non-developing RA first degree relatives (non-RA FDR), in FDR developing rheumatoid arthritis (FDR-RA), and newly diagnosed RA patients.

**Infection**	**Healthy controls (*n* = 283)**	**Non-RA FDR (*n* = 257)**	**FDR-RA (*n* = 26)**	**New RA (*n* = 283)**	**All**	**FDR-RA vs. Non RA FDR**	**FDR-RA vs. New RA**
Bronchitis acute	18 (6.4%) 2 (1–2.5) events 14 (8.5–18) days	20 (7.8%) 2 (1–3) events 14 (12–14) days	2 (7.8%) 4.5 (3–6) events 19.5 (18–21) days	30 (10.6%) 2 (1–3) events 14 (11–21) days	0.566 0.544 0.544	1 0.474 0.673	1 0.580 1
Bronchitis chronic exacerbation	4 (1.4%) 2 (1.25–2) events 45 (34–48) days	15 (5.8%) 2 (1–3) events 30 (17–30) days	3 (11.5%) 3 events 24 (21–30) days	24 (8.5%) 2 (1–3.75) events 27 (18–30) days	0.005 0.544 0.544	0.801 0.628 1	1 1 1
Herpes zoster	3 (1.1%) 1 events 14 days	2 (0.8%) 1 event 30 days	0– –	4 (1.4%) 1 event 13.5 (10–27) days	1 1 1	1 1 1	1 1 1
HSV reactivation/recurrence	67 (23.7%) 2 (1–3) events 7 (3–7) days	94 (36.6%) 2 (1–4) events 7 (7–10) days	12 (46.2%) 4 (1.5–6) events 7 (4–10) days	82 (29.0%) 2 (1–3) events 7 (5–10) days	0.012 0.235 1	1 0.309 1	1 0.371 1
Otitis acute	2 (0.7%) 2.5 (1–4) events 6.5 (3–10) days	7 (2.7%) 1 (1–2) events 14 (7–14) days	0 – –	20 (7.1%) 2 (1–4) events 10 (7–14) days	0.002 1 0.783	1 1 1	0.58 1 1
Otitis chronic exacerbation	0– –	2 (0.8%) 4 events 9 (4–14) days	0 – –	2 (0.7%) 2.5 (2–3) events 12 (10–14) days	1 1 1	1 1 1	1 1 1
Pneumonia	0 – –	1 (0.4%) 1 event 14 days	0– –	2 (0.7%) 1.5 (1–2) events 26 (21–30) days	0.845 1 1	1 1 1	1 1 1
Sinusitis acute	8 (2.8%) 1 (1–2) events 7.5 (7–14) days	8 (3.1%) 1.5 (1–3) events 12 (10–14) days	1 (3.8%) 1 events 21 days	12 (4.2%) 1 (1–3) events 14 (8–20) days	1 1 1	1 1 1	1 1 1
Sinusitis chronic exacerbation	4 (1.4%) 2.5 (1.25–3) year 15 (11–22) days	10 (3.9%) 3 (2–7.5) events 14 (13–23) days	2 (7.8%) 1 event 14 days	4 (1.4%) 3.5 (2.25–4) events 15.5 (14–27) days	0.145 0.290 1	1 0.309 1	1 0.446 1
Skin infection	4 (1.4%) 1.5 (1–2) events 10.5 (7–14) days	17 (6.6%) 1 (1–2) events 14 (10–20.5) days	5 (19.2%) 1 (1–7) events 12 (10–17.5) days	14 (4.9%) 1 (1–4) events 14 (14–20) days	< 10^−4^ 1 0.551	0.323 1 1	0.290 1 1
Stomatitis	1 (0.4%) 3 events 7 days	3 (1.2%) 3 (1–12) events 10 (10–14) days	0 – –	7 (3.8%) 1 (1–2) events 7 (6–30) days	0.334 1 1	1 1 1	1 1 1
Tonsillitis acute	39 (13.8%) 2 (1–3) events 5 (2–7) days	61 (23.7%) 3 (2–5) events 7 (4–10) days	12 (46.2%) 4 (3–5.75) events 10 (7.25–14) days	79 (27.9%) 3 (2–6) events 10 (7–14) days	< 10^−4^ 0.002 < 10^−4^	0.309 0.754 0.309	1 1 1
Tonsillitis chronic exacerbation	12 (4.2%) 5 (3–12) events 7 (3–10) days	39 (15.2%) 8 (4–12) events 10 (5–14) days	4 (15.4%) 9.5 (3–12) events 11.5 (8–13) days	20 (7.1%) 3 (2–5) events 14 (10–14) days	< 10^−4^ 0.002 0.242	1 1 1	0.628 0.371 1
Upper respiratory infection (URI)	161 (56.9%) 2 (1–2) events 6 (3–7) days	158 (61.5%) 2 (1–3) events 7 (4–7) days	14 (53.8%) 4 (3–6) events 7 (3.75–10) days	140 (49.5%) 2 (1–4) events 7 (4–10) days	0.122 < 10^−4^ 0.025	1 0.03 1	0.628 0.116 1
URI + antibiotics	32 (11.3%) 2 (1–2) events 7 (4–13) days	78 (30.4%) 4 (2–6) events 10 (4–14) days	6 (23.1%) 2.5 (1–5) events 17.5 (9–37) days	40 (14.1%) 2 (1–5) events 11 (7–20) days	< 10^−4^ < 10^−4^ 0.019	1 0.474 0.474	1 1 1
Upper urinary tract	3 (1.0%) 1 (1–3) events 14 (10–14) days	4 (1.6%) 2 (1.25–2) events 19 (12–28) days	0 – –	6 (2.1%) 2 (1–4.5) events 14 (14–21) days	1 1 1	1 1 1	1 1 1
Lower urinary tract	1 (0.4%) 1 events 10 days	7 (2.7%) 2 (1–2) events 10 (5–10) days	2 (7.8%) 6.5 (1–12) events 7 (4–10) days	4 (1.4%) 2 (1–5) events 10 (5.5–13) days	0.032 1 1	0.757 1 1	1 1 1
Hepatitis A virus (life)	7 (2.4%)	7 (2.3%)	0	1 (0.4%)	0.290	1	1
Hepatitis B virus (life)	3 (1.0%)	1 (0.4%)	0	0	0.560	1	1
Hepatitis C virus (life)	0	0	0	0	1	1	1
Tuberculosis in history	0	3 (1.2%)	3 (11.5%)	3 (1.0%)	< 10^−4^	0.309	0.261
Active tuberculosis	0	0/3	0/3	0/3	1	1	1
Pneumonia (life)	26 (9.2%)	28 (10.9%)	4 (15.4%)	20 (7.1%)	0.560	1	0.628
Presence of chlamydia, mycoplasma, ureaplasma	10/54 (18.5%)	14/47 (29.8%)	5/7 (71.4%)	14/33 (42.4%)	0.032	0.474	0.628

*The annual (or life when reported) infectious prevalence are reported and when positive the incidence per year (events: number of event/year) and the annual event duration (days: days/event/year) are expressed as median and interquartile range (IQR). The p-values corrected using the Benjamini–Hochberg method for multiple testing (58 tests) are presented. HSV, herpes simplex virus; URI, upper respiratory infections*.

### Infection prevalence in FDR-RA

**Figure 2:**
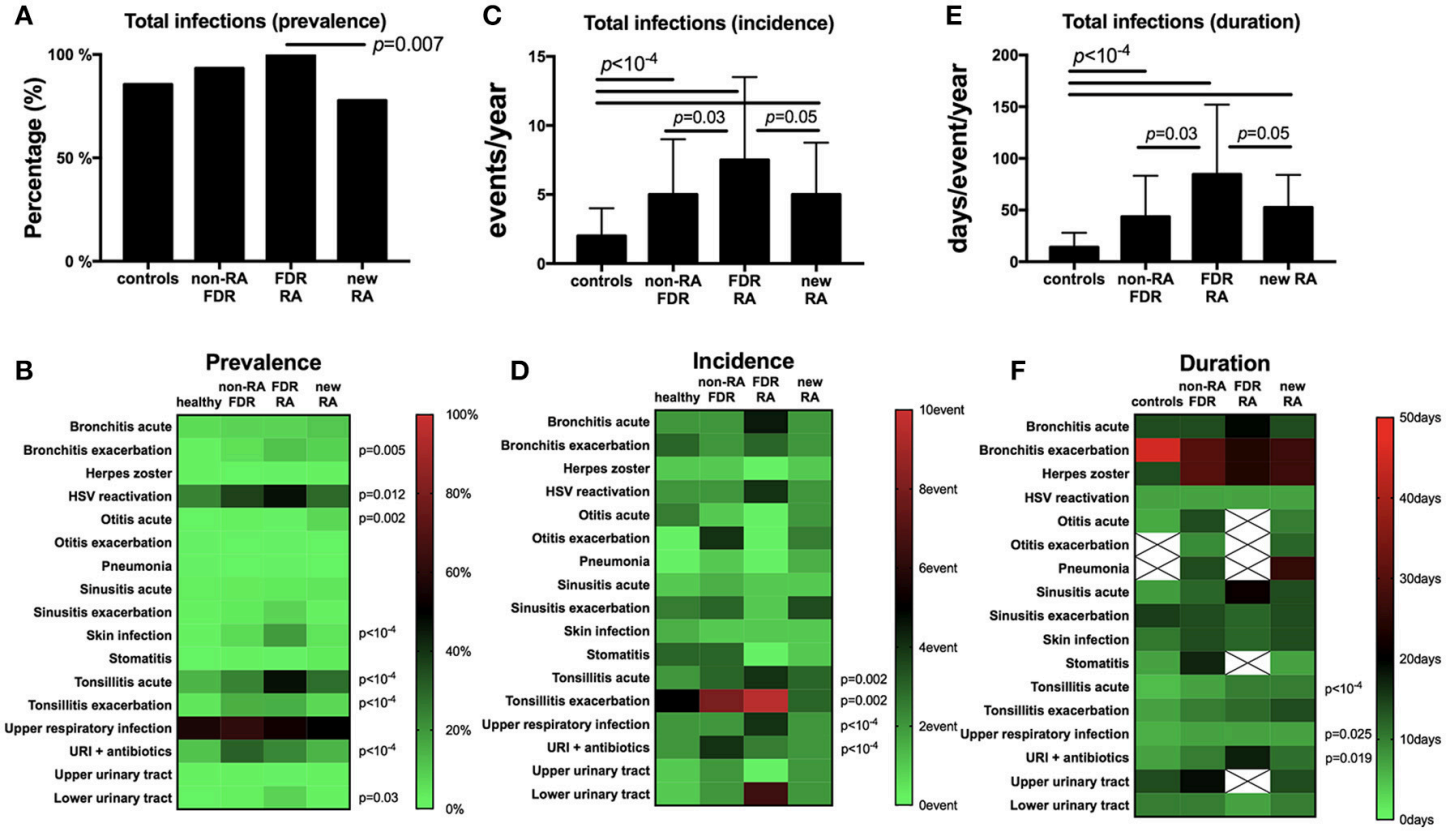
Infection prevalence, incidence and duration are increased in the FDR-RA subgroup at baseline. **(A,B)**: total **(A)** and individual **(B)** infection prevalence in the previous year in controls, non-RA FDR, FDR-RA and new RA. **(C,D)**: total **(C)** and individual **(D)** infection incidence (number of event/year). **(E,F)**: total **(E)** and individual **(F)** infection duration (day/event/year). new RA, newly diagnosed RA patients; FDR, first degree relatives; FDR-RA, FDR individuals having developed RA; FDR, individuals not having developed RA; URI, upper respiratory infections; HSV, herpes simplex virus infection. Hatched squares mean that no data were available. Statistics are indicated when *p* < 0.05 and for individual infections the *p*-values were corrected using the Benjamini–Hochberg method (58 tests).

At baseline (Figure 2A), total infection prevalence reported in the last year was different between the four subgroups (*p* < 10^−4^), and such differences were retrieved when comparing FDR-RA individuals with new RA patients (*p* = 0.007). Next (Figure 2B), individual infection prevalence was evaluated revealing differences related to bronchitis chronic exacerbations (*p* = 0.005), HSV exacerbation/reactivation (*p* = 0.012), otitis acute infections (*p* = 0.002), skin infections (*p* < 10^−4^), tonsillitis acute (*p* < 10^−4^) and chronic exacerbations (*p* < 10^−4^), URI receiving antibiotics (*p* < 10^−4^), and lower urinary tract infection (*p* = 0.03). In addition, an elevated report of tuberculosis in the history (*p* < 10^−4^), while activity was not confirmed using the quantiferon assay, and *Mycoplasma* sp. detection during pregnancy (*p* = 0.032) were also reported in FDR-RA individuals.

### Infection incidence and duration in FDR-RA

When regarding the incidence and the episode duration per year at baseline, significant differences were observed for both criteria when total infections were reviewed between subgroups (*p* < 10^−4^; Figures 2C,E). When comparing FDR-RA with non-RA FDR and with newly diagnosed RA subgroups, differences were conserved for both total infection incidence and the annual episode duration (*p* = 0.05).

Regarding individual infection incidences and durations (Figures 2D,F), differences were observed for tonsillitis acute (incidence: *p* = 0.002; duration: *p* < 10^−4^) and chronic (incidence: *p* = 0.002), and URI when untreated (incidence: *p* < 10^−4^; duration: *p* = 0.025) or treated with antibiotics (incidence: *p* < 10^−4^). When comparing RA-FDR with non-RA FDR individuals and newly diagnosed RA patients, only URI annual incidence was increased in RA-FDR (*p* = 0.03).

Altogether, we conclude from this cross-sectional analysis performed at baseline (i) that unaffected FDR-RA individuals have reported an elevated annual infection rate affecting predominantly the upper respiratory tract (URI, otitis, tonsillitis), herpes reactivation and skin infections; (ii) that differences are not only quantitative (prevalence) but also qualitative (incidence and to a lesser extent disease duration); and (iii) that infections are predominantly benign and recurrent.

### Infection events follow-up in FDR-RA

**Figure 3:**
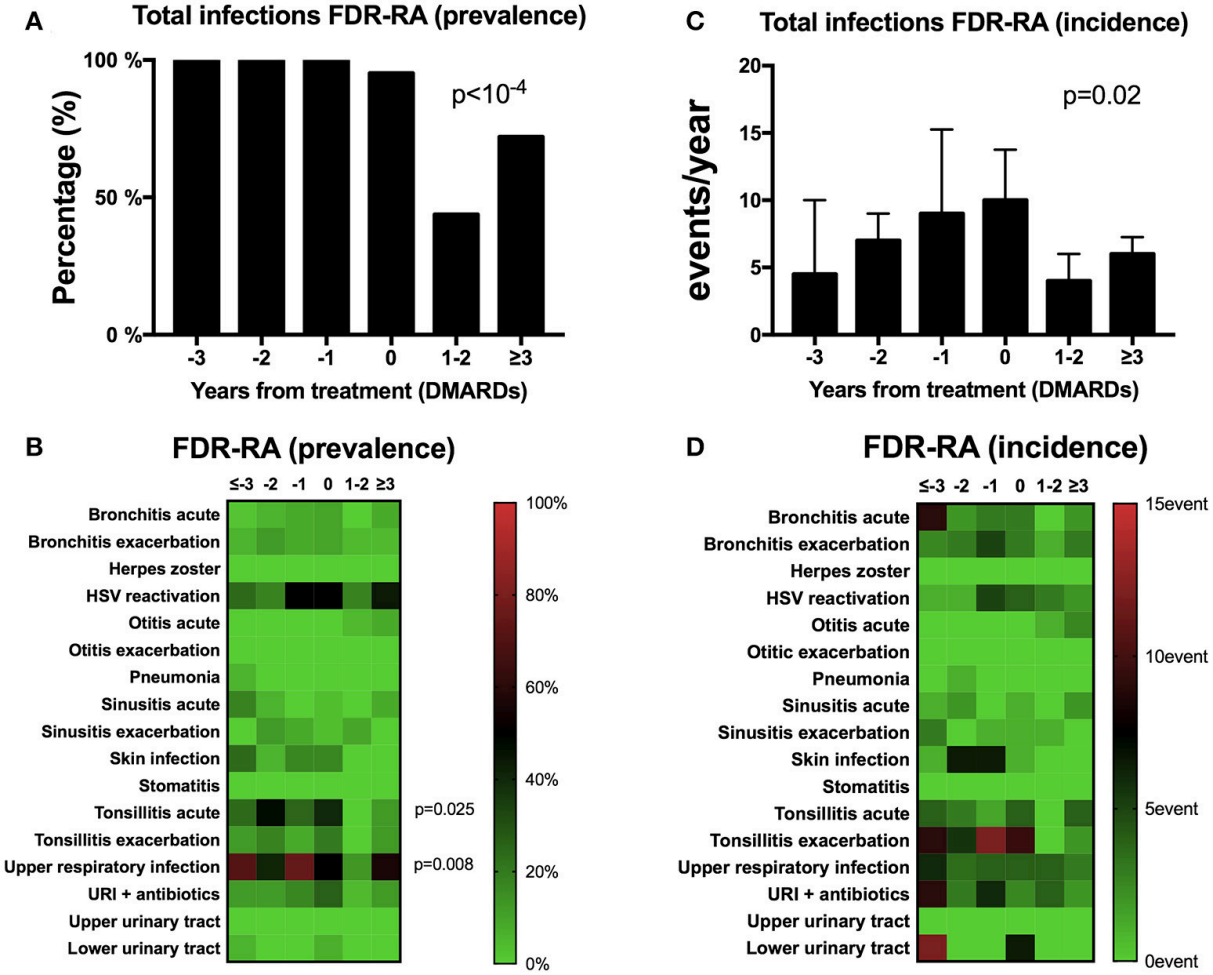
A transient peak of infection events is observed during the preclinical stage of RA. **(A,B)** total **(A)** and individual **(B)** infection prevalence from follow-up of the 26 first degree relative individuals having developed rheumatoid arthritis (FDR-RA); **(C,D)**: total **(C)** and individual **(D)** infection incidence (events/year). The time point is defined in relation to the diagnosis and DMARDs initiation (−3 or more years, −2 years, −1 year, 0 = diagnosis, +1/2 years and >3 years). new RA, newly diagnosed RA patients; FDR, individuals not having developed RA; URI, upper respiratory infections; HSV, herpes simplex virus infection; DMARDs, disease-modifying anti-rheumatic drugs. Statistics are indicated when *p* < 0.05 and for individual infections the *p*-values were corrected using the Benjamin–Hochberg method (17 tests).

As the prevalence and incidence of total infection events were significantly higher in FDR-RA individuals, we further suspected a peak of infection events preceding RA onset and/or therapeutic initiation with DMARDs. In order to answer this question, the infection events within the previous year were recorded from 26 FDR-RA individuals at different time points before RA diagnosis (−3 or more, −2, −1, and 0 years) and in the next 3 years following RA diagnosis and DMARDs introduction (+1/2, +3 years). As shown in Figure 3A, infection was the hallmark of all FDR-RA individuals in the years preceding RA onset while after RA diagnosis and DMARDs introduction a decrease was observed when considering total infection prevalence. Implicated infections were mainly related to URI (*p* = 0.008), and acute tonsillitis (*p* = 0.025; Figure 3B).

Regarding total infection incidence (Figure 3C), the total number of infection events/year increased gradually to the maximum within the last year preceding RA diagnosis and treatment (time point 0) and decreased after +1/2 years and ≥3 years of RA initiation (*p* = 0.02). Although not significant (Figure 3D), a similar trend was observed when considering individual infection incidence instead of prevalence.

### Dependence with clinical but not serological factors

**Figure 4:**
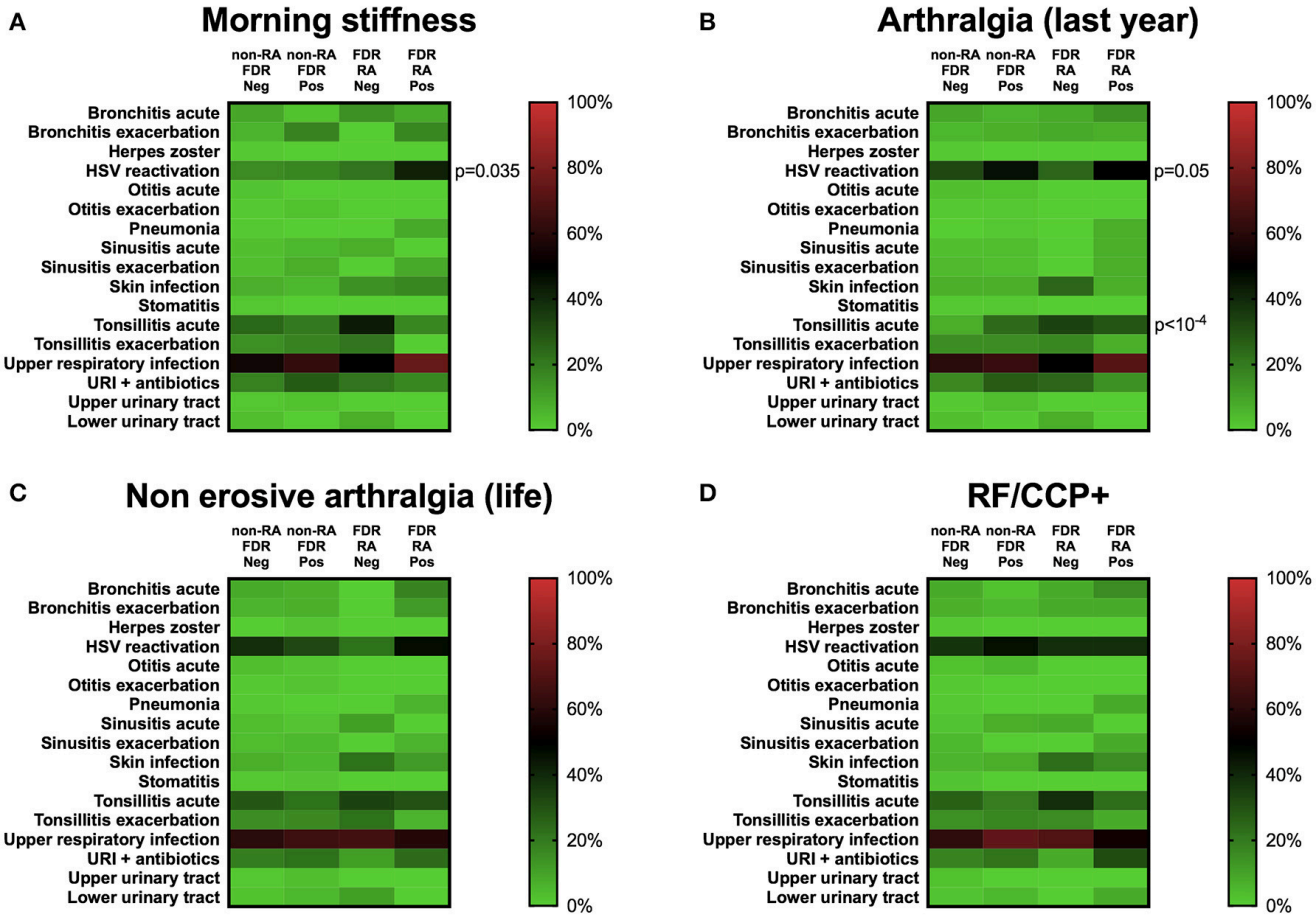
Herpes simplex virus (HSV) reactivation is associated with clinical but not with serological factors at baseline. **(A)** Morning stiffness (>30 min) in the previous year. **(B)** Arthralgia in the last year. **(C)** Non-erosive arthralgia in life. **(D)** Serological status regarding rheumatoid factor (RF) and anti-cyclic peptide citrullinated (anti-CCP2). Neg, subgroup negative for the clinical/biological factor tested; Pos, subgroup positive for the clinical/biological factor tested; FDR, first degree relatives not having developed RA during the follow-up; FDR-RA, FDR individuals having developed RA; URI, upper respiratory infections; HSV, herpes simplex virus infection. The *p*-values were corrected using the Benjamini–Hochberg method (17 tests) and statistics are indicated when *p* < 0.05.

Regarding clinical and serological factors associated with RA development (Table 2) and as previously described (22), non-erosive arthritis during life (*p* < 10^−4^), morning stiffness in the last year (*p* = 0.0006), and anti-CCP/RF positivity (*p* = 0.007) characterize FDR-RA patients at baseline from non-RA patients and even more from healthy controls (*p* < 10^−4^). To go further, we have tested an association with individual infection reports showing that morning stiffness (*p* = 0.035), and arthralgia in the previous year (*p* = 0.05) were associated with increased HSV reactivation prevalence, which predominated in FDR-RA individuals (Figure 4). Of note, no association was found when considering non-erosive arthralgia ever in life, and RF/CCP positivity. An association with acute tonsillitis was also associated with arthralgia (*p* < 10^−4^).

**Table 2:** Risk factors and clinical characteristics of unaffected first degree relatives (FDR) of probands with rheumatoid arthritis (RA) developing rheumatoid arthritis (FDR-RA) compared to healthy controls and FDR non-developing RA (non-RA FDR).

	**Healthy controls (*n* = 283)**	**Non-RA FDR (*n* = 257)**	**FDR RA (*n* = 26)**	**All**	**FDR-RA vs. Non RA FDR**
Age in years (median [IQR])	38 (22–53)	39 (26–51)	45.5 (34.5–55)	0.496	0.999
**JOINT SYMPTOMS AT BASELINE**					
Arthralgia last year	17/283 (6.0%)	85/257 (33.1%)	14/26 (53.8%)	< 10^−4^	0.082
Morning stiffness (>30 min) last year	0/283 (0%)	40/257 (15.6%)	12/26 (46.2%)	< 10^−4^	0.0006
Non-erosive arthritis (in life)	15/283 (0.4%)	62/257 (24.1%)	17/26 (65.4%)	< 10^−4^	< 10^−4^
Any joint symptoms (in life)	31/283 (11%)	129/257 (50.2%)	11/26 (42.3%)	< 10^−4^	0.609
Any small joint symptoms (in life)	3/283 (1.1%)	74/257 (28.8%)	3/26 (11.5%)	< 10^−4^	0.132
**SEROLOGY AT BASELINE**					
Negative RF and/or CCP levels	109/111 (98.2%)	163/204 (79.9%)	13/26 (50%)	
Low RF and/or CCP levels	1/111 (0.9%)	35/204 (17.2%)	12/26 (46.2%)	< 10^−4^	0.007
High RF and/or CCP levels	1/111 (0.9%)	6/204 (2.9%)	1/26 (3.8%)	
**ENVIRONMENTAL RA-ASSOCIATED RISK FACTORS**					
Smoker (no/passive/active)	62/20/14	37/11/2	14/2/0	0.193	0.480
Alcohol (no/rarely/regularly)	14/59/4	12/30/0	3/7/0	0.496	0.999
Body mass index	24.3 (20.4–29)	23.6 (20.3–27.6)	24.4 (20.5–30.5)	0.660	0.999
Fish consumption (no/yes)	17/58	11/29	1/7	0.660	0.583
Education (secondary/high/university)	32/50/11	28/37/4	6/8/0	0.660	0.999

*The p-values corrected using the Benjamini–Hochberg method for multiple testing (12 tests) are presented. CCP, autoantibodies to citrullinated peptides; IQR, interquartile range; NS, not significant; RF, rheumatoid factor*.

### Independence with environmental risk factors

Finally, non-infection environmental risk factors associated with RA development were explored in controls, FDR-RA and non-RA-FDR. No difference was observed between these subgroups regarding smoking activity, alcohol consumption, BMI, fish consumption as source of omega-3 and the educational level of the individuals. As a consequence, the absence of differences between subgroups supports the concept that infection events reported in this study are independent from these factors.

## Discussion

Results from this study highlight several points: (i) an elevated risk of developing RA is observed in FDR of women with RA from the Tatarstan women cohort; (ii) a transient peak of upper respiratory tract infection events in the preclinical period of the disease, which is probably controlled by DMARDs introduction; and (iii) an association between HSV reactivation and morning stiffness and arthralgia.

Among the 283 FDR individuals of probands with RA studied during this prospective 20-years longitudinal study cohort of Tatarstan women (mean duration 9 years), 26 developed RA, equivalent to an incidence of 9.1/1,000 per year which is similar to an incidence of 8/1,000 person-years reported by Silman et al. in a 10-years follow-up study (23), while others have reported only a small risk (as reviewed in 24). No cases have been reported in the healthy control group which is not surprising based on an incidence rate of RA for women to be 0.4/1,000 person-years in the healthy population (25), and to the fact that the follow-up was optional.

The window for studying infections during RA evolution is important and this is highlighted by the fact that we reported an elevated prevalence associated with a transient peak of infection incidence in the pre-clinical period of the disease (−2/0 years), and when RA diagnosis is established and DMARDs treatment initiated less infection events are observed. This is in line with the reports of important changes in the composition of the microbiome in mucosal surfaces during RA development in humans and rodents (26). One explanation, according to the immunological hypothesis, is related to the fact that, at the pre-clinical stage of RA in which T cells break tolerance, naïve CD4 and CD8 T cells are deregulated and have a distinct metabolic signature in order to hyperproliferate and acquire proinflammatory effector functions (27). These modifications have important consequences with defective capacity of the primary T cell responses to properly control viral and bacterial infections (28–30). The immunological hypothesis with a defective immune control of infections at the early stage of RA is further reinforced by the observations that granulocytes are altered in their capacity to induce reactive oxygen species and that B cell evolution to memory B cells is impaired (12, 31, 32). Similarly, the recent report from the Swedish population-based Epidemiological Investigation of early RA (EIRA) supports an insufficient Ab response at the early stage of RA with lower anti-viral IgG levels for EBV, CMV, and parvovirus B19 (10). If true such a hypothesis would reinforce our observations that differences regarding infectious events concern not only qualitative and immune independent (prevalence) parameters but also immune controlled parameters (infectious events incidence and/or duration). Interestingly, we also observed differences regarding site-specific infections with a higher risk in the upper respiratory tract that is counterbalanced with the lowered risk of RA associated with gastrointestinal and urogenital infections (33). At the opposite, arguments from our study supporting the infectious hypothesis are not as strong and are related to the higher prevalence of herpes reactivation and *Mycoplasma sp* observed in FDR-RA, two well-known infectious agent associated with arthralgia and detected in synovial fluids from RA patients (34, 35). A large longitudinal study that investigates direct interaction between infections and immune response is now required to further clarify whether infections are the cause or the consequence of immune aberrations reported in RA patients at the preclinical stage.

After treatment initiation with DMARDs to block T cell activation (36), infection events decreased in our cohort. Such an effect seems to be related to DMARDs introduction based on the RABBIT German cohort conclusions that has reported a lower prevalence of non-serious respiratory tract infections and skin infections in the first year following treatment initiation in the arm receiving DMARDs in contrast to the patients treated with tumor necrosis factor (TNF) inhibitors (37). This is further supported by the randomized control trials in which similar infection rates were reported between placebo and TNF inhibitor arms (38, 39). Accordingly, we propose that DMARDs introduction (but not TNF inhibitors) in RA patients makes them better regarding infections, this supports again the possibility that something about the immunology of the disease and the pre-disease states are driving the infection risk. At the opposite, arguments for infection being a trigger for RA are not as strong as in this case treating early RA patients prone to infection with immunosuppressors it would be expected that infections would worsen. To test such a hypothesis new experiments are needed that will explore the cellular mechanisms involved during the time course of the disease.

In accordance with the data of the Indigenous North American (INA) population presenting a familial RA disease aggregation (22), FDR individuals with RA development are more frequently seropositive (anti-CCP2 Abs ± RF in all cases) and present greater disease activity. In our study and during the pre-clinical phase of the disease, joint symptoms were not contributive as risk factors in contrast to morning stiffness, arthralgia and a history of non-erosive arthritis episodes that were present 2- to 3-fold more often in the FDR-RA subgroup in contrast to the non-RA FDR subgroup. Morning stiffness is considered to be a high risk factor useful for predicting RA (40, 41), and during RA, morning stiffness reflects disease activity with impact on the patient's quality of life. From the physiopathological point of view, morning stiffness is associated with inflammation of the synovium that surround tendons, referred to as tenosynovitis, rather than intra-articular synovitis as observed in other joint symptoms (42). Regarding factors associated with synovial inflammation we have observed an association restricted with HSV reactivation that is an attractive candidate as an overexpression of the herpes virus entry mediator (HVEM) in RA-FLS is suspected of contributing to HSV dissemination and to RA progression through pro-inflammatory cytokine production (43). Regarding non-infectious environmental factors and in contrast to other studies conducted in healthy FDR of probands with RA (44, 45), no association was found with smoking, primary educational level, BDI and fish consumption with controls.

This study has potential limitations and the first is related to the fact that such analysis is a single center study and lacks confirmation of non-serious infections; that all participants were women; and that information obtained from outpatient medical history specifies antibiotics used not bacterial agents except for urinary and genital tract infections. The relatively small number of FDR that develop RA is another limitation in terms of statistical power and general robustness and reproducibility of the findings presented, and particularly when reporting a small number of events for each reported infection. The low prevalence and the lack of any significant associations with tobacco and alcohol may result from a selection bias as the studied population was restricted to women and half were Muslims, who have low use of tobacco and alcohol. The socio-economic status of the individuals was not available and co-founder adjustments were not performed for environmental factors as data were not available for all individuals. Another limitation is related to the fact that RA-FDR individuals were probably more prone to seek care than non-RA FDR during their follow-up. To circumvent this limitation, comparison was performed at baseline before the FDR-RA patients fulfill RA criteria.

Regarding strengths, the current work is one of the first study comparing matched healthy individuals, non-RA and RA-developing individuals from an FDR population presenting high risk of developing RA at baseline and followed longitudinally for 20 years. The recruitment of FDR *via* patients with RA is another strength (46). Other strengths are related to the fact that such a study has investigated not only the exposure to infectious agents but also the annual number and the duration of all infection events at the preclinical and clinical stages of the disease.

In conclusion, a peak of infection events, RF/ACPA positivity, morning stiffness and non-erosive arthritis can be considered as relevant conditions associated with the development of RA in FDRs. More research is needed in order to better understand the cross-talk between infections and the numerous immune dysregulations reported during RA development.

## Author contributions

All authors listed have made a substantial, direct and intellectual contribution to the work, and approved it for publication.

### Conflict of interest statement

The authors declare that the research was conducted in the absence of any commercial or financial relationships that could be construed as a potential conflict of interest.
