# Supplementary material for: Prevalence and Incidence of Upper Respiratory Tract Infection Events Are Elevated Prior to the Development of Rheumatoid Arthritis in First-Degree Relatives
Source: Front Immunol. 2018 Nov 29;9:2771. doi: 10.3389/fimmu.2018.02771 (PMC6281887; doi:10.3389/fimmu.2018.02771)
Supplement: Supplementary file 1 [file Table_1.DOCX]

**Supplementary table 1:** Criteria used in the questionnaire to establish infectious episodes within the last year.

| **Infection** | **Criteria about the diagnosis** | **Questionnaire** | | | **Clinical confirmation** |
| --- | --- | --- | --- | --- | --- |
|  |  | Yes/No | Number | Delay | Yes/No |
| No infections during last year | Declared by the individual no one clinical manifestation of any infection within the last year. | Yes | - | - | - |
| Acute respiratory infections of the upper respiratory tract infection symptoms (URI) of multiple and unspecified site (ICD10*: X J06) | URI symptoms: catarrhal phenomena; not accompanied by itchy skin rashes in the form of urticaria, angioedema; gradually developing with a prodromal period in the form of increasing symptoms of malaise (intoxication-like feelings, low-grade fever, headache, myalgia, arthralgia); lasting 3-14 days (in cases not complicated by secondary bacterial infections); developed as a result of contact with an infected persons, as well as after general and local cooling, overheating, emotional / mental and physical stress (but not after known contact with the individual allergens or potential allergens or while receiving either of the drug); treatment carried out with antiviral, and optionally antipyretic drugs (but not antihistamines). | Yes | Yes | Yes | No |
| Herpes simplex type I/II virus (HSV) infection exacerbations (B00, A60) | HSV infection was evaluated by questioning about typical clinical manifestations (blisters mainly on the lip and nose mucosa after hypothermia events, lack of sleep, mental stress, the effect of local antiviral therapy).  In solitary cases of herpetic stomatitis or keratitis, or atypical dermal or mucosal localization of the blisters, the diagnosis was verified by the ear, nose, throat (ENT) doctor, dentist, dermatologist, or oculist based on the clinical, laboratory (serology) and instrumental examination.  In case of genital infections, the frequency and duration of HSV exacerbation events was assessed by a gynecologist. | Yes | Yes | Yes | Yes except blister |
| Herpeszoster (B02) | In all cases, the diagnosis was made by a general practitioner based on the clinical, laboratory (serology) and instrumental examination, and further confirmed by a specialist in infectious diseases. | Yes | - | - | Yes |
| Tonsilitis / Sinusitis / Bronchitis / otitis (acute and exacerbations of the chronic) (H60-H95, J00-J05, J42, J20) | The diagnosis was made by a general practitioner who treated the patient in uncomplicated cases. With a more severe / protracted episode and in all cases of exacerbation of the chronic infectious focus the diagnosis was verified by an ENT doctor.  The use of antihistaminic drugs was an exclusion criterion. | Yes | Yes | Yes | Yes |
| Pneumonia during the last year / Pneumonia ever in life (J18) | In all cases, the diagnosis was made by a general practitioner and confirmed in the therapeutic department of a regional hospital for treatment. | Yes | Yes | Yes | Yes |
| Upper and Lower urinary tract infections (acute and exacerbations of the chronic one) (N30, N34.0-34.2, N10, N11) | In all cases, the diagnosis was made by a general practitioner and was confirmed by the urologist who has examined and treated the patient. | Yes | Yes | Yes | Yes |
| Oral and odontical infections (K05) | Stomatitis, periodontitis was assessed by a dentist. | Yes | Yes | Yes | Yes |
| Skin and soft tissue infections (L00-L99) | Pyoderma, streptoderma, erysipelas, carbuncle/furuncle was assessed by a dermatologist or a surgeon (in case of surgical opening of carbuncle/ furuncle). | Yes | Yes | Yes | Yes |
| Bacterial complication of an upper respiratory tract infection requiring antibiotic therapy (B97) | Bacterial complication of a viral infection was determined if an acute respiratory infections of the upper respiratory tract (tonsils, ears, paranasal sinuses) in three to seven days was complicated by a purulent inflammatory process requiring antibiotic therapy (but not antihistamines, exclusion criterion). | Yes | Yes | Yes | No |
| VIRAL HEPATITIS in anamnesis (B15-B19) | In all cases the diagnosis was verified by a specialist in infectious diseases who examined and treated the patient based on the clinical, laboratory (serology) and instrumental examination. | Yes | - | - | Yes |
| Tuberculosis in anamnesis (A15-A19) | TB infection in anamnesis verified by TB specialist based on the clinical, laboratory (serology, Quantiferon assay) and instrumental examination. | Yes | - | - | Yes |
| Chlamydia, Mycoplasma, Ureaplasma infection at pregnancy (A60, A63.8, A49.3) | As laboratory diagnosis of Chlamydia, Mycoplasma, Ureaplasma infection is mandatory for all pregnant women and when planning pregnancy we also took into account asymptomatic carriage of these infections on the laboratory test data. Infectious episodes were assessed by appropriate specialists (gynecologists, ENT doctors, pulmonologists), | Yes | - | - | Yes |

**^*^Disease according codes to the International Classification of Diseases (ICD10)**
